# Retraction: Response of breast cancer cells and cancer stem cells to metformin and hyperthermia alone or combined

**DOI:** 10.1371/journal.pone.0323982

**Published:** 2025-05-05

**Authors:** The PLOS One Editors

Following the publication of this article [1], concerns were raised regarding results presented in Figs 1, 5, and 6. Specifically,

- The Fig 1C S6K panel of this article [1] appears similar to the Fig 2B S6K1 panel of [2] despite representing different experimental conditions.
- When adjusting colour levels to visualise the background, there appear to be vertical irregularities in the following panels:
  - Fig 5B MCF-7 Cyclin D1 panel, between lanes 4 and 5
  - Fig 5B MDA-MB-231 Cyclin D1 panel, between lanes 4 and 5.
- The Fig 6A 37°C panels (0 mM, 0.5 mM, 1 mM, and 5 mM) of this article [1] appear similar to the Fig 4A panels of [2].

The corresponding author HJP stated that the Fig 1C S6K panel presented in this article [1] is incorrect. They provided the original data underlying the results presented in Fig 1C, including the original data for the correct S6K results.

The corresponding author HJP commented that the Fig 5B Cyclin D1 panels present spliced results that were prepared by combining data obtained from a short exposure blot (lanes 1-4) and a long exposure blot (lanes 5-8).

The corresponding author HJP confirmed that the same control data (37°C) were reported in Fig 6A of [1] and Fig 4A of [2] and stated that the raw FACS data underlying Fig 6 are no longer available. In light of these concerns, the corresponding author HJP requested retraction of this article [1].

The *PLOS One* Editors retract this article due to the unresolved concerns with Fig 6A, which call into question the reliability of the reported results and conclusions about cancer stem cells.

HJP agreed with the retraction. HL, CSP, ETO, BHC, BW, CKL, and CWS either did not respond directly or could not be reached.

The Fig 1C S6K panel and the Fig 6A 37°C panels present material previously reported in [2], published in 2012 by Song et al. under a CC BY-NC-SA 3.0 license. Due to restrictions that apply to the original article’s [2] license, these panels are excluded from this article’s [1] license. The article [1] was republished on April 21, 2025, to note these exclusions in the Fig 1 and Fig 6 legends and the article’s copyright statement.
